# VirAmp: a galaxy-based viral genome assembly pipeline

**DOI:** 10.1186/s13742-015-0060-y

**Published:** 2015-04-28

**Authors:** Yinan Wan, Daniel W Renner, Istvan Albert, Moriah L Szpara

**Affiliations:** 1 The Huck Institutes of the Life Sciences, University Park, PA 16802 USA; 2 Department of Biochemistry and Molecular Biology, Pennsylvania State University, University Park, PA 16802 USA

**Keywords:** Next generation sequencing, herpes simplex virus, viral genome, assembly pipeline, variation analysis

## Abstract

**Background:**

Advances in next generation sequencing make it possible to obtain high-coverage sequence data for large numbers of viral strains in a short time. However, since most bioinformatics tools are developed for command line use, the selection and accessibility of computational tools for genome assembly and variation analysis limits the ability of individual labs to perform further bioinformatics analysis.

**Findings:**

We have developed a multi-step viral genome assembly pipeline named VirAmp, which combines existing tools and techniques and presents them to end users via a web-enabled Galaxy interface. Our pipeline allows users to assemble, analyze, and interpret high coverage viral sequencing data with an ease and efficiency that was not possible previously. Our software makes a large number of genome assembly and related tools available to life scientists and automates the currently recommended best practices into a single, easy to use interface. We tested our pipeline with three different datasets from human herpes simplex virus (HSV).

**Conclusions:**

VirAmp provides a user-friendly interface and a complete pipeline for viral genome analysis. We make our software available via an Amazon Elastic Cloud disk image that can be easily launched by anyone with an Amazon web service account. A fully functional demonstration instance of our system can be found at http://viramp.com/. We also maintain detailed documentation on each tool and methodology at http://docs.viramp.com.

**Electronic supplementary material:**

The online version of this article (doi:10.1186/s13742-015-0060-y) contains supplementary material, which is available to authorized users.

## Findings

### Background

Recent assembler evaluations such as GAGE [1] and Assemblathon 2 [2] have indicated that parameter tuning and adapting the assembly process to match properties of the genome are essential steps for obtaining high quality assemblies. This demonstrates the need for tools that provide customizable pipelines that life scientists can run repeatedly to evaluate the effects of the various parameters on the quality of the assembly. In this paper we present VirAmp, a virus assembly pipeline designed to process high coverage shotgun sequencing data obtained from virus genomes. VirAmp combines into a single Galaxy interface [3] a set of existing tools and best practices that facilitate straightforward multistep, semi *de novo* assembly approaches.

Advances in high-throughput sequencing make it possible to sequence a large number of viral genomes at high coverage even in a single sequencing run. At the same time viral genomics presents scientists with a number of unique challenges and requires tools and techniques developed specifically to account for the much faster mutation and recombination rates that these genomes typically exhibit [4,5]. As a consequence, there is a high demand for tools that can efficiently perform various analysis tasks commonly associated with viral assemblies. Detecting variation by mapping against a reference genome is a frequently used methodology when studying higher order eukaryote genomes. This strategy is appropriate for the analysis of SNPs, small insertions and deletions (indels), and mutations that involve only a few bases. Due to faster mutation rates, short generation times, and more intense selective pressures, viral genomes may be genetically distant from the known reference genomes. *De novo* assembly solves some of these challenges at the cost of added algorithmic and computational complexity. Caveats of *de novo* assembly include the uncertain nature of gaps and the condensed size of short sequence repeats, which are assembled at the most compact size supported by the data. However these caveats are outweighed by the ability of *de novo* assembly to detect regions that alignment cannot, such as large insertions or rearrangements, and sequences that diverge significantly from prior reference genomes.

There are multiple approaches to *de novo* assembly. *Overlap-layout-consensus*, or OLC, uses multiple sequence alignment (MSA) to orient and connect the short sequence reads and produce a final consensus sequence [1,6]. This approach works well for Sanger sequencing data, but it is less well suited for next-generation sequencing data which commonly consists of much more numerous, but shorter sequencing reads. In contrast, the *de Bruijn graph*-based algorithms assemble data by representing the genome via a set of short subsequences (or k-mers) [1,7]. For these algorithms the sub-sequence size (k-mer size) becomes an essential parameter of the process. Contigs (or extended sequences built by overlapping reads) that are created using a data representation of short k-mers tend to be smaller but contain fewer errors. In contrast, contigs built from longer k-mers can reconstruct repeats more precisely, but at the cost of introducing minor errors and variations that can lead to gaps or breaks in the final assembly. *De Bruijn* graph construction is non-deterministic, in that it depends on the order of sequence reads, however this rarely affects the performance or downstream analysis. In general, assemblies generated from *de Bruijn* graph based assemblers tend to contain smaller contigs compared to those obtained from *overlap-layout-consensus* algorithms.

The constrained size of viral genomes, along with the increasing yield of sequencing instrumentation and methods, have combined to give researchers extremely high rates of coverage when sequencing viral genomes using this approach. While theoretically this high coverage is not needed, in practice it may be necessary so that a sufficient amount of data is obtained from hard-to-sequence regions of the genome, such as areas with high G + C content or secondary structures. As a consequence, the coverage of a single base of a viral genome may vary from tens to tens of thousands of reads. This radical variability in read coverage introduces specific algorithmic challenges, as most tools and techniques were not designed to handle data with such properties. Methodologies such as digital normalization [8] have been introduced to reduce redundant information in deep sequencing data. In this paper we demonstrate that by combining several existing approaches and techniques we can produce nearly complete high quality viral assemblies in less than two hours on a single CPU computer with 4 GB of memory. We validated our pipeline using sequencing data from both laboratory and clinical strains of HSV-1, which represent a wide range of variation with respect to the reference genome of HSV-1, including SNPs, indels, and short sequence repeats (SSRs) that are present in many viral genomes.

### Assembly pipeline description

**Figure 1:**
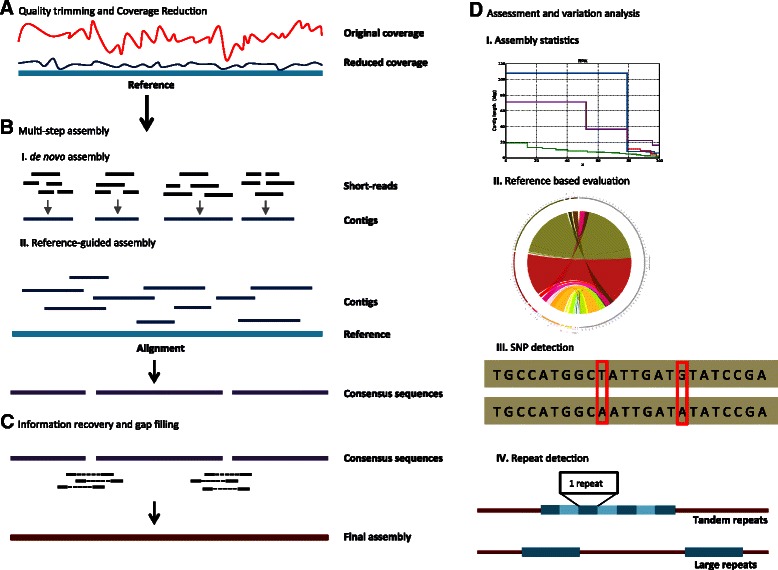
VirAmp pipeline overview. The diagram illustrates the progression of the VirAmp pipeline. **A)** First, we perform a quality trimming of the raw data, then reduce extremely high coverage data (top trace, red) to a reasonable depth and even out the coverage variation (bottom trace, blue; usually to ~100x). **B)** Next, a multi-step semi-*de novo* strategy is applied for core assembly: (I) a *de novo* assembler is run multiple times using different k-mer sizes, to assemble the short sequence reads into a set of long contigs; (II) contigs from different k-mer sets are oriented by aligning to the reference genome and then are connected into scaffolds based on the pairwise alignment. **C)** Data from the spacing of paired-end reads is used to extend the contigs, potentially closing gaps and/or joining contigs into larger scaffolds. **D)** Multiple tools are implemented for assembly evaluation and analysis of variation. These include basic assembly statistics, comparison of the new assembly to a reference genome, and identification of SNPs and repeats.

The VirAmp pipeline consists of a series of connected analytical methods that were found to be necessary for optimal assembly of viral genomes. As shown in Figure 1, the main steps consist of: 1) quality control of input data, 2) coverage reduction, 3) *de novo* genome assembly, 4) reference-guided genome assembly, 5) information recovery and gap-filling, and 6) quality evaluation of final genome assembly. Additional optional steps include 7) final gap closing, 8) assembling single-end sequence reads, and 9) additional ways to access the VirAmp pipeline. Below we discuss in more detail the rationale for each step:

1. **Quality control of input data.** Various artifacts and errors inherent to the sequencing process may affect the data obtained from a sequencing instrument. Correcting these may require various trimming and filtering steps that remove unreliable sections of the data. In our pipeline the default trimming is performed via the seqtk toolkit [9], which implements the Phred algorithm and is able to remove low-quality bases from the end of a sequence read. In addition, we provide a collection of optional quality control tools that offer functions such as polyA and adaptor clipping, as well as base quality trimming. We also provide tools to filter data for contaminating sequence reads derived from the host cell genome, using Bowtie2 [10] as the underlying aligner. Users may choose between the various tools and apply the quality filtering before sending the data into later steps of the pipeline. Detailed documentation is provided for each of these tools.
2. **Coverage reduction.** The highly variable coverage inherent to deep sequencing of viral genomes poses specific challenges to most genome assemblers. Reads that fall into very high coverage areas do not provide new information, yet they can negatively affect the performance of the algorithms. Since these redundant reads may also be affected by sequencing errors, they may increase the dimensionality of the data, further reducing the efficiency of the assembly process. To correct for redundant coverage we integrate a digital normalization step via the **diginorm** [8] approach into our pipeline. Digital normalization is a computational algorithm that uses k-mer abundance to estimate the sequence depth of the unassembled genome. Diginorm systemizes the coverage of short reads, discards redundant reads, and reduces the impact of sampling variation and sequencing errors. Digital normalization will reduce coverage to a predefined cutoff while retaining most of the reads covering low coverage regions.
3. ***De novo*genome assembly.** Our pipeline operates via a two-step strategy that integrates different assembly methods, thus benefiting from information produced by *de novo* assembly protocols as well as reference guided multiple sequence alignment algorithms (Step 4 below). This allows us to capture a larger number of variations than using either method separately. After coverage reduction via diginorm, our pipeline uses *de novo* assembly to place the short reads into longer blocks of continuous sequence called contigs. Because the assembly output depends on the choice of k-mer size, we run multiple rounds of *de novo* assembly with different k-mers, and then combine them into a single dataset that becomes the input for the next step in the pipeline. Our default installation offers three commonly used assemblers:
  - **Velvet** [7] is one of the earliest assemblers using the *de Bruijn* graph algorithm. It is designed as a general assembler for shotgun sequencing. Velvet is set as our default choice for *de novo* assembly.
  - **SPAdes** [11] is an assembler designed for standard isolates and single-cell Multiple Displacement Amplification (MDA) bacterial assemblies. SPAdes uses an iterative approach to implement a multisized *de Bruijn* graph algorithm with multiple k-mer sizes. SPAdes is also available as a complete pipeline, but here we use only the core assembler.
  - **VICUNA** [4] is an OLC algorithm-based *de novo* approach that specifically targets assembly of virus genomes with a high mutation rate. This tool can handle deep sequencing data with high variation, at the cost of potentially longer runtimes. As for SPAdes, only the core assembler of VICUNA is used here.
4. **Reference-guided genome assembly.** Once we obtain contigs from the *de novo* assembly step, the VirAmp pipeline will further orient and connect them into a draft genome using the reference-guided assembler **AMOScmp** [6]. AMOScmp uses an *alignment-layout-consensus* algorithm to orient the short contigs by aligning to a reference genome. AMOScmp then connects the short contigs together into a new draft genome by using information from a round of multiple sequence alignment. This algorithm is a modified version of the traditional OLC algorithm, which was originally designed for Sanger sequencing [12].
5. **Information recovery and gap filling.**
  - **Scaffold extension and connection with SSPACE.** To ensure that no information has been discarded at this stage, VirAmp makes use of a tool called SSPACE for further scaffolding and contig extension [13]. SSPACE is a stand-alone scaffolding tool, which we implement using the un-normalized input data to provide as many sequences as possible for assembly correction and expansion. SSPACE begins by using BWA [14] to align paired-end or mate-pair sequence reads back to the contigs assembled by AMOScmp. SSPACE can then extend these contigs by searching for unmapped reads whose mate-pair is located near the edge of a gap, and estimating the placement of these paired reads into the gap region(s). SSPACE then uses the spacing between paired-end reads to scaffold contigs together, forming longer stretches of intact sequence for the final genome assembly. SSPACE accounts for any information loss during the digital normalization and coverage reduction, since it extends and connects the contigs using the complete original dataset.
  - **Single linear sequence creation.** A final assembly with a set of ~5-10 contigs is created upon the completion of SSPACE. These contigs are listed in the order that they align to the reference genome, producing a linear genome that may contain several gaps. An optional step is provided to connect the contigs into one sequence by adding Ns to represent ambiguous bases between contigs. In this case, the number of Ns is estimated from the spacing found in the reference genome.
6. **Quality evaluation of final genome assembly.** To help researchers better understand and interpret their viral genome assembly results, we provide utilities for genome assessment and variation discovery.
  - **Assembly evaluation metrics via QUAST.** QUAST [15] is a quality assessment tool for evaluating genome assemblies. QUAST uses the MUMmer [16] aligner to analyze the newly assembled genome and compute reference-based and reference-free metrics. Important statistics such as contig number, N50 and NG50 are provided as part of this summary. N50 and NG50 are common metrics for comparing how well different assembly methods work for a given genome or dataset. To compute these statistics, all contigs are placed in order from longest to shortest. The sum of all contig sizes is recorded as the maximum possible assembly length (since duplicate and overlapping contigs exist, this is almost always longer than the target genome). Moving in order from longest to shortest, the N50 statistic represents the size of the contig at which half the maximum assembly length has been achieved. Large N50 values reflect assemblies with large contigs, without an excess of small contigs. NG50 is very similar to N50, except that the comparison is to the reference genome length. A large NG50 value indicates that a majority of the reference genome length is encompassed by contigs of this size or longer, which is beneficial for the quality of the final assembly. A full version of the QUAST report is provided for users’ further exploration.
  - **Assembly-reference comparison.** The assembly-reference comparison report provides details about the alignment of the newly assembled genome against the viral reference genome. Coordinates and percent identity are provided for each aligned region between the two sequences. This helps the user to identify large indels, as well as other complex structural variations. Table 1 demonstrates an example of the comparison report.

**Table 1:** **Overview of the assembly-reference comparison**

Ref_start	Ref_end	Contig_start	Contig_end	% Identity	References	Contigs
1	62457	288	62722	99.46	JN555585_truncated	Ctg_1
53191	53334	53597	53454	100.00	JN555585_truncated	Ctg_1
62638	108009	62722	108094	99.15	JN555585_truncated	Ctg_1
108111	108301	186	1	92.75	JN555585_truncated	Ctg_1
108299	108496	5	193	92.96	JN555585_truncated	Ctg_2
108497	116585	296	8423	97.10	JN555585_truncated	Ctg_2
116652	116924	8421	8687	93.04	JN555585_truncated	Ctg_2
117090	117383	9718	9042	86.50	JN555585_truncated	Ctg_2
117497	117634	9044	9181	100.00	JN555585_truncated	Ctg_2
117897	123237	1	5261	97.37	JN555585_truncated	Ctg_3
123245	134543	1	11337	98.74	JN555585_truncated	Ctg_4
134593	136376	1	1754	97.42	JN555585_truncated	Ctg_5
  - **Circos graphs.** Circos [17] is a software package that is used to visualize data in a circular layout. Our pipeline produces a circular graph as part of the assembly-reference comparison report (Figure 2). The right-hand side of the circle contains a linear representation of the reference genome, and the new draft genome is displayed as a set of ordered contigs on the left side of the circle. Arcs connect the contigs of the draft genome on the left, to the matched sections of the reference genome on the right. Circos provides a visual overview of the alignment between the draft genome and the reference genome.

**Figure 2:**
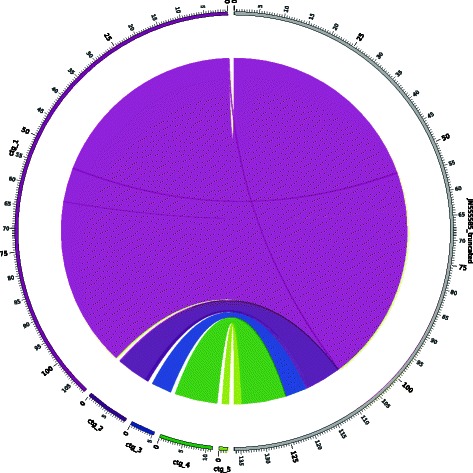
Assembly-reference comparison via Circos graph. Circos graphs can be used to compare between an assembled genome and a reference genome. Here we present the comparison of a newly assembled draft HSV-1 genome containing five scaffolds on the left semicircle (colored bands), to the HSV-1 reference genome (NCBI JN555585) on the right semicircle (grey band). Each color represents one assembled scaffold, and the grey band represents the reference genome. The gaps between scaffolds on the left indicate the breakpoints between contigs that could not be joined by the VirAmp algorithms. These breakpoints indicate insufficient information, which could result from insertions, inconsistent information about overlaps between two contigs, or regions that could not be assembled. Note that the length of the gap remains the same for each breakpoint; this does not represents the length of an actual gap. Each tick mark represents 0.5 kb, with labels included every 5 kb, and bold type every 25 kb.
  - **Variation analysis.** VirAmp provides a collection of tools built upon the MUMmer [16] package for variation identification. SNP analysis produces a list of SNPs as a VCF (Variant Call Format) file. Structural repeats and tandem repeats can also be identified using tools we provided. BWA [14] is used to map the sequence reads back to the new assembly, which offers a means to verify the new assembly, and to detect minor variations that may reflect polymorphisms in the genome sequencing data.
7. **Final gap closing.** The end result of running our pipeline is an assembly built from multiple long contigs. Users have the option to generate two versions of each genome. The first of these is a multi-fasta file that usually contains a small number of contigs in the order and orientation that they align to the reference genome. We also produce a second file that contains only one linear genome sequence, which is generated by inserting Ns into the gaps between subsequent contigs, so that the linear draft genome closely corresponds to the reference genome. We note that automated gap closing may greatly oversimplify the complexities of genomic rearrangements. Its use should be restricted mainly to situations where a single linear genome sequence is necessary, such as sequence alignment between multiple strains. Gaps between each contig should be assessed carefully before closing. We recommend using the multi-fasta file for assembly assessment and variation discovery, since this reflects the most accurate outcome of the assembly process.
8. **Assembling Single-end Reads.** The use of paired-end sequence read data is strongly recommended when performing genome assemblies, because the larger insert sizes allow the algorithms to better infer positional location in the genome. However we have also implemented an alternative assembly pipeline for single-end reads. In this pipeline, the SSPACE scaffolding is not used, since it depends on the paired-end information to connect contigs. All other modules are utilized in the single-end pipeline (diginorm, velvet/SPAdes/VICUNA and AMOScmp).
9. **Additional ways to access the VirAmp pipeline.** The easiest path to utilize VirAmp is via the fully functional demonstration website at http://viramp.com/. All the modules and components of the VirAmp pipeline come pre-installed and integrated into a customized version of Galaxy [3]. Galaxy is an open source, web-based platform that provides a web interface for commonly used bioinformatics tools. This facilitates use by researchers without programming experience. Users can also choose to launch their own VirAmp instance via an Amazon Elastic Cloud machine image (AMI) that can be easily launched by anyone with an Amazon web service account. Support and updates to VirAmp are documented in a GitHub repository (http://github.com/SzparaLab/viramp-project).

**Figure 3:**
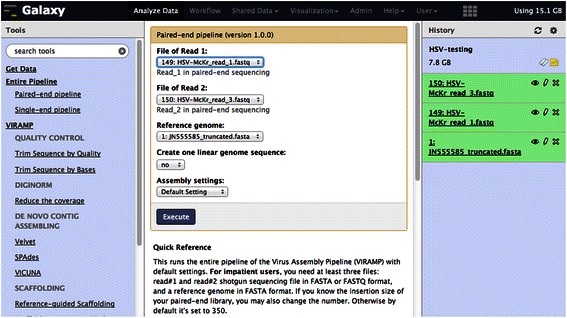
View of VirAmp input page. The VirAmp pipeline is presented in a Galaxy-based interface. Drop-down menus allow users to select input files and carry out the entire pipeline using default settings. A full menu of component steps is available on the left, for advanced users who want to run or tune individual steps.

VirAmp offers the ability to run a complete viral genome assembly pipeline in a single step, with the required inputs being only the raw FASTQ format data files and a reference genome from a related species in FASTA format. The pipeline will then output the major results and visualizations. We provide interfaces to operate each step separately, so that those familiar with assembly tools can select and tune individual steps. The program is hosted via the Amazon Elastic cloud and we provide a customized AMI that other labs can launch to serve their individual computational needs. The disk images are fully customized and ready to run upon launching; these require no additional system management to operate. We provide detailed documentation on how to start a custom version of VirAmp at: http://docs.viramp.com. A ready-to-use demonstration instance of the VirAmp pipeline is also available at http://viramp.com/ (Figure 3).

### Viral sequence inputs to VirAmp

Viral genome sequencing data usually originates from one of two approaches. The first involves targeted sequencing of viral isolates of interest, which often entails expansion of the virus population using host cells or a host animal. This approach generates large quantities of viral genetic material where the contaminating host sequences are identifiable and fairly homogeneous. In contrast, the second common type of viral genome sequencing stems from field or clinical samples. In this approach the viral genome being sought is not the majority genome present and there may be multiple, or even hundreds, of genomes from other species contributing to the mix of nucleic acids. VirAmp accepts sequence reads from either approach, and the quality of the assembly output will reflect the purity and quantity of viral sequence reads provided as input. Removal of contaminating host or environmental sequence reads will facilitate assembly of the target viral genome. Although we have developed VirAmp for assembly of large (typically DNA-based) virus genomes, RNA virus genomes that have been reverse-transcribed for library preparation can also be used as input.

To generate clean, paired sequencing reads for optimal *de novo* assembly, it is important to remove sequences that stem from low-quality base calls, technical artifacts, or host genome contamination. This filtering can entail the removal of whole sequence reads, or just the trimming of bases from one end. Because the removal of host sequences is usually virus and host-cell specific, we recommend tools for this approach but cannot provide a universal approach with all possible host genomes pre-loaded. The sample data included at the http://viramp.com/ demonstration website has already been filtered using the following approach.

We followed previously published approaches to prepare HSV-1 DNA for sequencing [18,19]. Each viral isolate was expanded using a cultured cell line, in this case African green monkey kidney epithelial cells (ATCC® CCL-81™Vero cells). The viral DNA was isolated using a previously described procedure to enrich for viral DNA that is packaged in nucleocapsids [18,19]; this method generally produces sequencing libraries that contain 10% or less contaminating sequence reads from the host genome. Then, the FASTX-Toolkit was utilized to remove or trim technical artifacts such as library adapter sequences, fully monomeric reads, low quality bases, and sequences below a length minima (http://hannonlab.cshl.edu/fastx_toolkit/). Next, we used a bowtie alignment [10] to compare all sequencing reads against the rhesus macaque (*Macaca mulatta*) genome and removed any perfect matches. This genome was the best available match to the host Vero cells used to grow these HSV stocks. A final check removed any reads missing their paired-end sequencing mate. Parameters for these approaches have been previously described [18,19].

### Pipeline evaluation

We evaluated our protocols by assembling data obtained from the genome of HSV-1. HSV-1 is one of the most prevalent human pathogens, infecting around 70% of adults worldwide. In most cases it causes mild epithelial lesions, but the virus remains infectious for a lifetime, with sporadic recurrences that allow spread to new hosts [20]. The reference strain HSV-1 17 has a genome of 152 kb (GenBank Accession JN555585). The genome consists of a 108 kb unique long (UL) and a 13 kb unique short (US) region, with each unique region flanked by inverted copies of large structural repeats (termed repeat long (RL) and repeat short (RS), with lengths of 9.2 kb and 6.6 kb) [21]. For evaluation purposes, we used a trimmed version of this reference where the terminal copies of RL and RS have been removed, leaving a sequence of 136 kb (Figure 2). The removal of terminal repeats facilitates alignment of *de novo* assembled contigs to the reference genome. For evaluation, we used three datasets of 100 bp × 100 bp paired-end reads sequenced by Illumina protocols. Each dataset contained more than 30 million reads with an average genome coverage of over 10,000-fold. The observed average library fragment size without adaptors was 350 bp.

**Figure 4:**
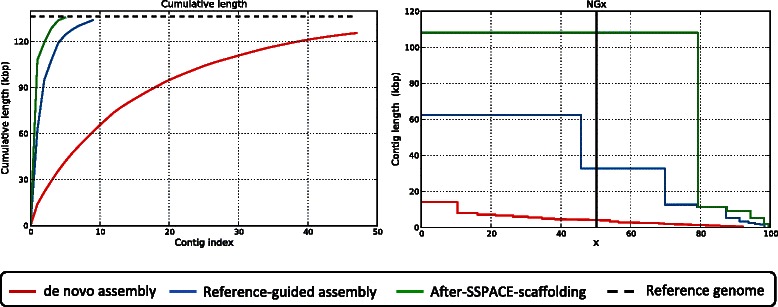
Statistics of assembly at each step of VirAmp. Cumulative data plots outputted by the QUAST package provide a visual overview of individual assembly steps, for a laboratory strain of HSV-1 (Table 2). Successive contigs are plotted in order from longest to shortest. In both graphs, the red line represents the output of the initial *de novo* assembly, the blue line represents the combination of multiple k-mer assemblies using reference-guided assembly approaches, and the green line represents the output after scaffolding by SSPACE. **A)** The first graph highlights the number of contigs (contig index, x-axis) needed to achieve the length of the trimmed reference genome (y-axis; 135 kb); this metric improves with successive steps of the VirAmp pipeline. Only contigs longer than 500 bp were considered to be valid. **B)** The second graph plots the percent of the genome (x-axis) covered as successive contigs are added, from longest to shortest. The y-axis intersect for each line is the length of the longest contig, and the line drops according to length of each successive contig. The black vertical line indicates NG50. The total length, largest contig, and NG50 all increase with each step of the VirAmp pipeline.

To demonstrate the necessity and contribution of each stage of the pipeline, we performed a QUAST assessment [15] at every step of the process instead of just at the conclusion of the process, using data for a laboratory strain of HSV-1 (Table 2). Figure 4 shows the basic statistics from assembly evaluation of each step of the VirAmp pipeline. We used the NG50 statistic as our metric since as demonstrated in Assemblathon 2, it is a more appropriate parameter than N50 when a reference genome is present [2]. NGx is an extension of NG50, where x represents the percent of reference genome bases encompassed by the contigs (e.g. NG50 means 50% of reference genome). Velvet was used for the *de novo* assembly step with multiple k-mer sizes (k = 35,45,55,65). The statistics for this step (Figure 4, red line) represent the best assembly of the above four sets (k = 65). With each successive step of the VirAmp pipeline the ability of the contig collection to minimally tile the viral genome improved (Figure 4A). Additional assembly metrics improved as well, such as the summed length of all contigs, the length of the largest contig, and the NG50 (Figure 4B). After scaffolding (Figure 4B, green line) the largest contig covered about 80% of the reference genome, which is more than 108 kb out of 136 kb (Table 1).

**Table 2:** **Performance comparison using different assembly pipelines**

Virus^1^	# reads (x 10^6^)	Pipeline	# contigs	Largest contig (bp)	N50	NG50	# fully un-aligned contigs	REAPR score	Run time (h)	# thread (4GB/CPU)
HSV-1 **laboratory strain**	33	VirAmp	5	108,094	108,094	108,094	0	1.28e + 10	1.5	1
		SPAdes	9,609	107,857	258	107,857	9582	4.12e + 7	6	4
		VICUNA	266	19,285	5,654	8,704	163	6.01e + 8	8	6
HSV-1 w/ **fluorescent insert**	37	VirAmp	4	63,109	49,971	49,971	0	4.00e + 9	2.5	1
		SPAdes	5,946	39,441	273	13,888	5898	4.48e + 7	7	4
		VICUNA	101	33,391	9,822	7,644	60	9.03e + 8	13	6
HSV-1 **clinical isolate**	87	VirAmp	3	117,134	117,134	117,134	0	1.24e + 9	4	1
		SPAdes	74,927	93,771	256	82,041	74,608	2.97e + 8	21	4
		VICUNA	424	23,611	2,786	7,136	383	2.19e + 8	30	6

^**1**^Bold indicates abbreviation used in text, figures, and Additional file 1.

We examined the starting and ending coordinates of selected contigs from each step of the HSV-1 laboratory strain assembly (Additional file 1). From this inspection, we observed whether the gaps -- missing bases between the ending coordinates of one contig and the starting coordinates of the next -- had been narrowed or closed in each step (Additional file 1). We found that both the reference guided assembly step (AMOScmp) and the scaffold extension and gap-filling step (SSPACE) decreased the total number of gaps and narrowed the remaining gaps. From thousands of contigs produced by initial *de novo* assembly (Velvet), AMOScmp reduced this complexity to less than fifteen contigs and SSPACE yielded a total of just five contigs that spanned the reference genome with only minor gaps (Figure 2 and Additional file 1). By using digital normalization before assembly, followed by using the full dataset for extension and scaffolding after the assembly, we were able to integrate the most amount of information from the sequencing data into the assembly with reduced computational resource usage.

Mapping the final assembly set of the laboratory HSV-1 strain back to the HSV-1 reference genome revealed few overlaps between the contigs and suggested that this is an almost linear genome (Figure 2). VirAmp provides an option to connect these contigs into one linear genome, which may be helpful for downstream functional analysis.

### Comparing the performances of VirAmp with other assembly pipelines

To assess the performance of our assembly pipeline, we used three different HSV-1 sequencing datasets for evaluation. We selected datasets from a virulent HSV-1 laboratory strain, a variant laboratory strain with a fluorescent protein inserted into the genome, and a clinical isolate of HSV-1. These datasets contain from 33 to 87 million Illumina HiSeq reads of paired-end, 100 bp × 100 bp sequence (Table 2). Using previously published approaches [18,19], we de-multiplexed these sequence reads, trimmed off adaptor sequences, removed low quality bases, removed sequencing artifacts, and removed sequences matching the genome of the host cells used for growing viral stocks. The Utilities menu of VirAmp includes tools for these steps, but we did not incorporate these into the default pipeline because we anticipate user-customization at this phase (e.g. whether or not to de-multiplex, choice of host genome, etc.). We used the above datasets to compare our Velvet-based pipeline with two other standalone assembly pipelines, SPAdes [11] and VICUNA [4]. SPAdes is a pipeline optimized for genome assemblies on the bacteria scale. The SPAdes pipeline includes an error correction preprocessing step as well as mismatch correction as a post-assembly process. Its core assembler can make use of multiple k-mer sizes, taking advantage of both small and large k-mers to improve the assembly performance. Single-cell mode was applied in SPAdes using the authors’ recommended k-mer sizes (k = 21, 33, 55). The VICUNA pipeline is an alternative *de novo* assembly pipeline developed by the Broad Institute specifically for virus genome assembly. One of the advantages of VICUNA is that it performs a pre-filtering step to keep only reference-genome-like reads, which is extremely useful in host-contaminated samples such as viruses. We performed multiple rounds of VICUNA assembly and chose the best k-mer (k = 21) for this comparison. The core assemblers in both SPAdes and VICUNA have been integrated into our pipeline so that end-users may choose either one as alternatives to the default Velvet assembler.

We compared the assemblies back to the trimmed HSV-1 reference genome (136 kb), and used N50, NG50 and REAPR [22] scores to evaluate the performance of each assembly method, as recommended by Assemblathon 2 [2] (Table 2). We considered any contigs longer than 500 bp as a valid assembly output. All basic statistics except REAPR are calculated using a complete version of the QUAST [15] report generated from our pipeline at the end of the assembly. The additional metric used here, REAPR, is a reference-free tool to evaluate the genome assemblies [22]. This tool maps the paired reads back to the assemblies to evaluate accuracy per-base and per-scaffold. The REAPR score here was computed using version 1.0.16 under default settings, except for setting the mapping option –y to 0.9. The overall REAPR produces a score integrating three metrics: error free bases, original N50 and broken N50. This score summarizes aspects of local accuracy, overall assembly performance, and structural correctness at the scaffold level.

According to the evaluation statistics, the VirAmp pipeline achieves the highest NG50 and REAPR score in all three HSV-1 datasets (Table 2). In two of the three datasets the largest VirAmp contig covered about 75% of the whole genome. SPAdes retrieved one large contig with a length similar to the longest contig of VirAmp, but in all three test datasets more than 95% of the SPAdes contigs cannot be properly aligned back to the reference. This causes SPAdes to receive the lowest N50 and REAPR score among the three assemblers. VICUNA retrieved an assembly with a size similar to the reference and an acceptable number of contigs, but the largest contig it produced was only around 20kb, which is much shorter than the other two assemblers.

In terms of computational resources, VirAmp analyzed the above datasets on a single 4 GB RAM CPU machine while neither SPAdes nor VICUNA could finish the job successfully using the same machine. For a dataset with ~20,000-fold coverage on average (e.g. HSV-1 lab strain, Table 2) VirAmp finished the assembly within 1.5 hours, while the other two assemblers ran the same dataset with multiple CPUs (4 for SPAdes and 6 for VICUNA) with 4 GB RAM and took more than double the time to complete.

## Conclusion

In this paper we describe a web-based virus genome assembly platform, VirAmp, which can be used to assemble high throughput sequencing data. Our pipeline makes use of several existing programs and connects them in a convenient interface. The pipeline makes use of recommended practices and can assemble extremely high coverage viral genome data with minimal computational resources. In addition, we provide a series of reporting and genome assembly analysis tools for evaluating the assemblies. All of our tools are wrapped into a Galaxy instance that individual groups can utilize at the demonstration website or run independently. The Galaxy platform and default pipeline will facilitate use by researchers without advanced programming skills, or without access to high-performance computing clusters.

## Availability and requirements

**Project Name:** VirAmp: A Galaxy-based virus genome assembly pipeline

**Project Homepage:**http://viramp.com/

**Operation System:** Linux

**Programming language**: Python, Bash

**Other requirements:** None to use demonstration website or install using GitHub repository; Amazon web service account to launch own AMI

**License:** MIT License

**Any restrictions to use by non-academics:** None

## Availability of supporting data

All tools described, as well as testing datasets, are available at the VirAmp demonstration website: http://viramp.com/. A GitHub repository is available for the present AMI and all future updates: http://github.com/SzparaLab/viramp-project. The VirAmp project is available via GitHub at https://github.com/SzparaLab/viramp-project/. The specific commit SHA at the time of publication is 5e8aaef12192165718c66d4919ed21bb308a4600. Detailed documentation for using VirAmp or for launching a new AMI is found at: http://docs.viramp.com. Help notes are also embedded within each VirAmp tool. Within VirAmp, sample data is located under “Shared Data → Data Libraries”, including sequence read data for the three HSV-1 strains listed in Table 2. A smaller fourth dataset containing a quarter million reads of HSV-1 is also included for instant testing of the VirAmp pipeline. These data are also hosted at the *GigaScience* Database [23].

A workflow has been published under “Shared Data → Published Workflows”. Two sample histories have been published under “Shared Data → Published Histories”, both of which use the HSV-1 lab strain dataset. The first of these, “workflow-pe-hist”, was run with the published workflow while the other, “entire-pipeline-pe-hist”, was run with the prepackaged pipeline (“Entire Pipeline → Paired-end pipeline”). Due to the non-deterministic nature of *de novo* assembly described above, the results from each run of the pipeline may vary slightly, normally within 10 bp.

## Additional file

Additional file 1:
**(Word document) Table of contig coordinates for step-wise assembly of an HSV-1 laboratory strain, in comparison to the HSV-1 reference genome.**

## Abbreviations

HSV: Herpes simplex virus
INDEL: Insertions and deletions
MSA: Multiple sequence alignment
OLC: Overlap-layout-consensus
SNP: Single Nucleotide Polymorphism
VCF: Variant call format
VirAmp: Viral genome assembly pipeline
