# Supplementary material for: VirAmp: a galaxy-based viral genome assembly pipeline
Source: Gigascience. 2015 Apr 28;4:19. doi: 10.1186/s13742-015-0060-y (PMC4410580; doi:10.1186/s13742-015-0060-y)
Supplement: Additional file 1: — (Word document) Table of contig coordinates for step-wise assembly of an HSV-1 laboratory strain, in comparison to the HSV-1 reference genome. [file 13742_2015_60_MOESM1_ESM.docx]

Additional file 1.

Contig coordinates for step-wise assembly of an HSV-1 laboratory strain, in comparison to the HSV-1 reference genome.

| Ref_start | Ref_end | Contig_start | Contig_end | Contig_ID |
| --- | --- | --- | --- | --- |
| **Contig coordinates after scaffold extension (SSPACE)** | | | | |
| 1 | 108009 | 288 | 108094 | Ctg_1 |
| 108111 | 117634 | 5 | 9181 | Ctg_2 |
| 117897 | 123237 | 1 | 5261 | Ctg_3 |
| 123245 | 134543 | 1 | 11337 | Ctg_4 |
| 134593 | 136376 | 1 | 1754 | Ctg_5 |
| **Selected contig coordinates after reference-guided assembly (AMOScmp)** | | | | |
| 75278 | 108004 | 14 | 32695 | AMOSctg_3 |
| 108111 | 108281 | 9 | 174 | AMOSctg_4 |
| … … | | | | |
| 117476 | 117634 | 1 | 159 | AMOSctg_10 |
| 117897 | 119760 | 1 | 1864 | AMOSctg_11 |
| 1119846 | 123229 | 1 | 3387 | AMOSctg _12 |
| 123251 | 134537 | 1 | 11325 | AMOSctg_13 |
| 134595 | 136376 | 1 | 1752 | AMOSctg_14 |
| **Selected contig coordinates after *de novo* assembly (velvet)** | | | | |
| 101276 | 108004 | 6723 | 1 | NODE_25 |
| 109208 | 109807 | 1 | 597 | NODE_45 |
| … … | | | | |
| 115768 | 116021 | 252 | 1 | NODE_50 |
| 120582 | 120928 | 347 | 1 | NODE_52 |
| 121397 | 122313 | 1 | 915 | NODE_34 |
| 123396 | 128815 | 4 | 5429 | NODE_21 |
| … … | | | | |
| 131850 | 134423 | 1 | 2579 | NODE_32 |
| 134595 | 136376 | 1791 | 40 | NODE_26 |

Ascending order of coordinates for reference genome locations, with the corresponding contigs to the right of each reference location.
